# A DNA Damage Response-Independent Mechanism for Telomere Shortening-Elicited Age-Related Pathologies

**DOI:** 10.1093/geroni/igaa057.3268

**Published:** 2020-12-16

**Authors:** Amanda Stock, Kun Wang, Chongkui Sun, Chengyu Liu, Yi Gong, Yie Liu

**Affiliations:** 1 National Institute on Aging, Bethesda, Maryland, United States; 2 National Heart, Lung, and Blood Institute, Bethesda, Maryland, United States

## Abstract

Telomere attrition is associated with telomeropathies and age-related pathologies. In telomeropathies, telomere uncapping induces a DNA damage response (DDR) that drives apoptosis or senescence. However, a defined mechanism by which telomere attrition contributes to other age-related pathologies has not been determined. Telomere integrity is maintained by shelterin, a six-protein complex. Rap1 is the only shelterin member that is not essential for telomere capping but engages non-telomeric DNA and regulates gene transcription. We hypothesized that non-telomeric Rap1 accumulation could contribute to age-related pathologies in a DDR-independent manner. To test this, we used CRISPR/Cas9 editing to generate a Rap1 mutant mouse model in which Rap1 at telomeres is prevented, leaving only non-telomeric Rap1. Indirect immunostaining showed no differences in telomere dysfunction-induced DDR foci in Rap1 mutant compared to wild-type primary fibroblasts. Cell fractionation/western blotting of fibroblasts from Rap1 mutants demonstrated decreased Rap1 expression and Rap1 re-localization off telomeres, which mimics the same alteration of Rap1 in human cells with telomere attrition. Rap1 mutant mice exhibited increased body weight and altered metabolic and immune-response transcripts in various tissues, indicating that altered transcription could account for some of the observed phenotypes related to telomere attrition. In conclusion, telomere shortening may facilitate non-telomeric Rap1, which alters gene transcription and drives metabolic and immune dysfunction in a DDR-independent manner.

